# Molecular Investigation of *Theileria* and *Babesia* Species in Domestic Mammals from Sardinia, Italy

**DOI:** 10.3390/vetsci10010059

**Published:** 2023-01-14

**Authors:** Valentina Chisu, Elisa Serra, Cipriano Foxi, Giovanna Chessa, Giovanna Masala

**Affiliations:** Department of Animal Health, Istituto Zooprofilattico Sperimentale della Sardegna, Via Vienna, 2, 07100 Sassari, Italy

**Keywords:** *Babesia bigemina*, cattle, horse, *Theileria annulata*, *Theileria equi*, *Theileria orientalis*

## Abstract

**Simple Summary:**

*Babesia* and *Theileria* are tick-borne parasites in the Phylum Apicomplexa with considerable impact on animal and human health globally. In this study, 152 blood samples collected from Sardinian mammals were analyzed by PCR and sequencing targeting the 18S rRNA gene specific for the *Babesia*/*Theileria* species. *Babesia bigemina* and *Theileria orientalis/sergenti/buffeli* were detected in the cattle, while the DNA of *Theileria equi* was found in the horses. In addition, the presence of *Theileria annulata* was demonstrated for the first time in cattle from Sardinia. These results improve the epidemiological knowledge of piroplasm infections in cattle and horses from the study area and highlight that additional research is warranted to determine the prevalence of *Babesia* and *Theileria* infections in Sardinia.

**Abstract:**

Piroplasmoses are tick-borne diseases caused by hemoprotozoan parasites of veterinary and public health significance. This study focuses on the molecular identification and characterization of species belonging to the *Theileria/Babesia* genera in 152 blood samples, collected from 80 horses and 72 cattle from several farms in Sardinia, by targeting the 18S rRNA gene. The PCR results highlighted that 72% of the samples were positive for *Theileria/Babesia* spp., with a rate of infection of 68% and 75% for the horses and cattle, respectively. Sequencing and the BLASTn analysis showed that the 18S rRNA generated in this study has 99–100% homology with the *B. bigemina*, *T. orientalis/sergenti/buffeli*, *T. equi* and *T. annulata* strains isolated from different hosts worldwide. These findings improve the knowledge on *Babesia* and *Theileria* infections in domestic mammals and confirm the significant prevalence of piroplasmosis among subclinical and carrier animals throughout the island. Furthermore, the presence of *T. annulata*, reported for the first time in the study area, expands the repertoire of pathogens already detected in Sardinia. Our results gather updates on the diversity and distribution of piroplasms in Sardinia and suggest the need to develop procedures to improve animal and public health safety.

## 1. Introduction

Tick-borne hemoparasitic infections are major public health, veterinary and socio-economic burdens worldwide [1]. Among them, the piroplasm protozoans of the *Theileria* and *Babesia* genera (phylum Apicomplexa, order Piroplasmida) are responsible for economically important diseases in both domestic and wild animals [2]. Although asymptomatic infections are most common, piroplasmoses in animal hosts can result in severe anemia, weakness, fever and hemoglobinuria. Some species of *Babesia* can cause severe disease in humans [3], with symptoms that can range from flu-like symptoms to severe disease, with a pathogenicity and virulence that mainly depend on the infected animal host, its response to infection, the infecting protozoal strain and the inoculum size [4]. Human infections with *Theileria* strains are sporadic, and few cases of human theileriosis have been reported around the world [5,6]. Ixodid ticks of the genera *Amblyomma, Haemaphysalis, Hyalomma* and *Rhipicephalus* are involved in the transmission of the *Theileria* and *Babesia* species that parasitize the blood cells of mammalian hosts and infect a wide variety of domestic and wild vertebrates [7]. 

In particular, *Babesia* sporozoites infect the capillaries of vertebrate hosts during tick feeding. Once inside the erythrocytes, the *Babesia* spp. develop into round, oval or ring-shaped trophozoites that undergo asexual multiplication, in which pear-shaped merozoites are released from the erythrocytes and infect new erythrocytes. Hemolytic anemia and hemoglobinuria are caused by the destruction of erythrocytes [2]. By contrast, the *Theileria* spp. undergo sporogony in the tick salivary glands. After the tick bite, the sporozoites invade the host leukocytes. Merozoites are then released into the bloodstream and invade the erythrocytes [2].

Although different *Theileria* (namely *T. equi*, the *T. orientalis* group and *T. ovis*) and *Babesia* (*B. bigemina*) species have been reported in ticks collected from wild and domestic mammals, and recent data demonstrated the presence of *T. ovis*, the *T. orientalis* group and *B. major* in domestic ruminants from Sardinia [8,9], there is still a gap of information on the epidemiology of these hemoprotozoans on the island. On this premise, to gain further insight into the occurrence of piroplasmosis in Sardinia, this study reports the molecular detection and characterization of *Theileria* and *Babesia* strains.in cattle and horse blood samples on the island.

## 2. Materials and Methods

### 2.1. Sample Collection

**Figure 1:**
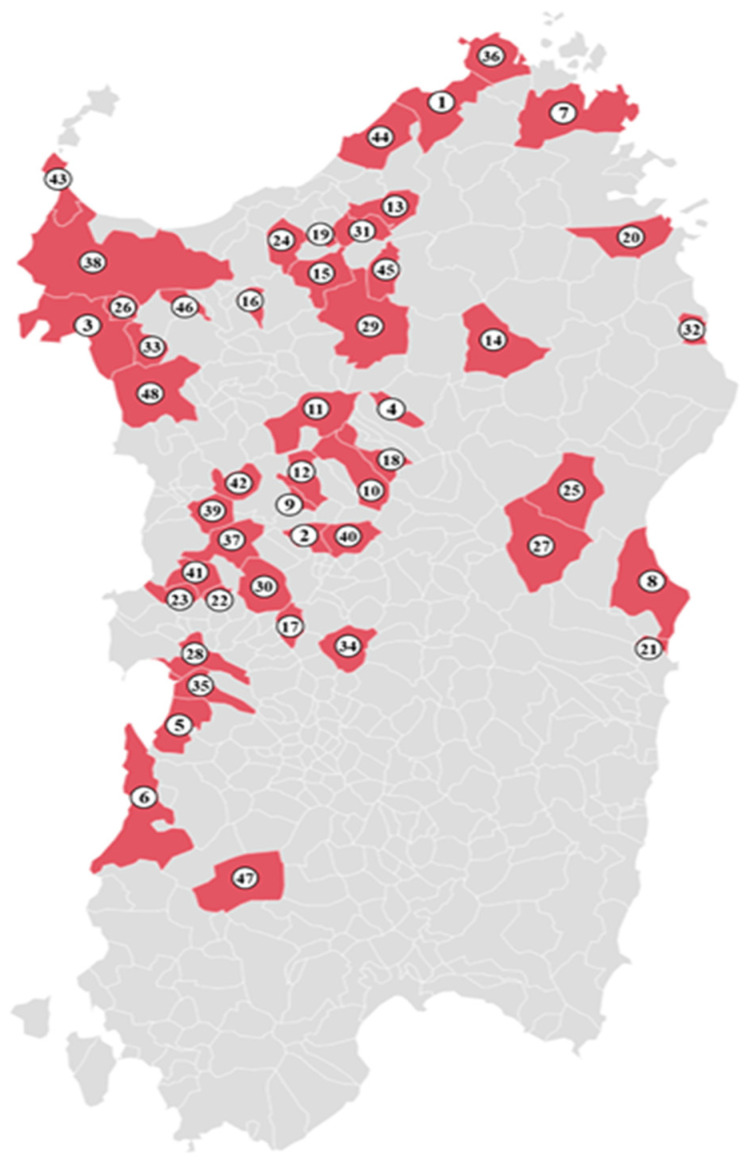
Map of Sardinia with sample collection sites for *Babesia*/*Theileria* molecular detection in mammals between 2020–2021.

From June 2020 to July 2021, 152 blood samples were randomly collected from cattle (*n* = 72) and horses (*n* = 80) from 48 farms (Figure 1) in Sardinia, the second biggest island of the western Mediterranean. This region shows high rates of ticks throughout the year, with rates intensifying during the late spring and summer months. The blood samples were taken from the jugular vein by sterile syringe, collected into EDTA-Vacutainer tubes and stored at 4 °C until the DNA extraction. 

### 2.2. DNA Extraction and Screening of Piroplasmida by PCR Amplification

Total genomic DNA was extracted from 200 µL of the EDTA blood sample using the DNeasy Blood and Tissue Kit, Qiagen (Hilden, North Rhine-Westphalia, Germany), according to the manufacturer’s instructions. The extracted DNA was frozen at −20 °C until further processing. A screening for *Babesia*/*Theileria* species was performed by PCR amplification using genus-specific primers, BJ1 (5′-GTCTTGTAATTGGAATGATGG-3′) and BN2 (5′-TAGTTTATGGTTAGGACTACG-3′), targeting a 469 bp fragment of the conserved region of the ribosomal 18S gene (18S rRNA) [10].

The PCR assay conditions were applied according to previously published methods [8]. Specifically, the PCR reaction mix was made up to a final volume of 25 μL and contained 12.5 μL of Amplitaq Gold Master Mix (Quantitect Probe PCR Master Mix, Qiagen, Hilden, Germany), 1 μL of 25 μM of each primer [10], 9.5 μL of RNase nuclease-free water and 1 μL of genomic DNA. A DNA control extracted from *B. bovis* and distilled water were used as the positive and negative controls, respectively. The thermal cycle conditions were 95 °C for 15 min for the initial denaturation, followed by 40 cycles of denaturation at 94 °C for 1 min, annealing at 60 °C for 30 s and elongation at 72 °C for 1 min. The amplification was completed by a further 5 min step at 72 °C. The PCR products were run on 1.5% agarose gel stained with SYBR^®^ Safe DNA Gel Stain (Invitrogen, Carlsbad, CA, USA), separated through horizontal electrophoresis and visualized on an Image Master transilluminator. The sizes of the PCR products were compared to a 100 bp DNA marker (Sigma-Aldrich Pty Ltd, Darmstadt, Germania).

### 2.3. Purification and Sequencing

The PCR-positive amplicons were then purified using the QIAquick PCR Purification Kit (Qiagen, Hilden, Germany), according to the manufacturer’s protocols. The purified products were sequenced in both directions using an ABI Prism BigDye Terminator Cycle Sequencing Ready Reaction Kit (Applied Biosystems, Foster City, CA, USA). The electropherograms obtained were assembled and corrected using ChromasPro software (ChromasPro 1.7, Technelysium Pty Ltd., Tewantin, Australia), and the nucleotide sequences were submitted and compared with those available in the GenBank database using NCBI BLASTn (http://blast.ncbi.nlm.nih.gov/Blast.cgi (accessed on 19 October 2022)).

## 3. Results

### Prevalence of Pathogens

The PCR results showed that 72% of the analyzed samples were positive for piroplasm DNAs, using PCR and sequencing of the 18S rRNA gene. Specifically, 49/72 (68%) and 60/80 (75%) of the blood samples collected from cattle and horses, respectively, tested positive for piroplasmid DNA. To determine the identity of the 109 positive samples detected by the 18S rRNA PCR, the samples were directly sequenced using the same forward and reverse primers as above. The results revealed that 65% (70/109) of the nucleotide sequences generated clear electropherograms that allowed for detecting and discriminating among the *Babesia*/*Theileria* species. Unfortunately, the chromatograms from the remaining PCR-positive samples did not generate a clear sequencing signal and were unreadable.

The BLASTn results of the 18S rRNA gene sequences allowed us to obtain four sequence types, as shown in Table 1. All the genotypes were associated with the *Babesia*/*Theileria* genus. In particular, the 18S rRNA sequences generated from horses were mostly similar (99–100%) to the *Theileria* strains recorded in GenBank as *T. equi* (Accession Number: MT463613), while the BLASTn analysis of the 18S rRNA target gene of the genotypes detected from bovines showed 100% identity with *T. orientalis* (Accession Numbers: MN611176 and MH208641), *T. annulata* (Accession Number: MT341858) and *B. bigemina* (Accession Number MH050356) strains isolated from various hosts worldwide.

## 4. Discussion

In this study, a molecular survey on piroplasmid infections was carried out in domestic mammals from Sardinia, Italy. The DNA of *Babesia* and *Theileria* has been previously detected in ticks collected from domestic and wild hosts [8] and from the blood of symptomatic and asymptomatic mammals (horses, sheep, cattle and domestic pigs) sampled from limited districts of north-western Sardinia [9]. In this study, four different *Babesia*/*Theileria* spp. were detected, including *T. annulata*, which was first identified in cattle from this region. A validated method consisting of PCR and sequencing targeting the 18S rRNA gene was used to detect and identify *Babesia* and *Theileria* DNA in blood samples collected from Sardinian mammals. Moreover, the 18S rRNA gene, due to the presence of a conserved and hypervariable region, proved useful as a marker to determine evolutionary patterns and similarity among the members of piroplasmids detected here. However, it should be pointed out that, since the identification of the piroplasms was conducted by using 18S rRNA partial nucleotide fragments, further study is needed for a definitive identification using more marker genes. 

The diversity of the piroplasms identified here could be related to the distribution of ixodid ticks in Sardinia. It has been known that ticks belonging to the *Haemaphysalis*, *Hyalomma* and *Rhipicephalus* genera are proven vectors for the transmission of *Babesia* and *Theileria* spp. [11]. All these tick species have been described in Sardinia, where significant differences in the levels of abundance and distribution among the tick species from different study areas have been reported [8]. In particular, we observed that a high prevalence of the *T. orientalis* group was detected in cattle from northern areas, while *T. annulata* was more frequent in western Sardinia. *B. bigemina* was detected in three of the cattle from northeastern Sardinia, while *T. equi* was detected in horses from several collection sites throughout the region. 

Almost half of all the cattle from this study were infected with *T. orientalis* (a member of the so-called *T. sergenti/T. buffeli/T. orientalis* group), which was the most frequently detected *Theileria* species (Table 1). *T. orientalis* is the causative agent of oriental theileriosis, a disease that has a clinical and economic impact on the cattle industry worldwide, especially in the Asia-Pacific region [12]. *T. orientalis* infection in cattle is most often asymptomatic, but mild symptomatic forms due to erythrocyte destruction are associated with anemia and hypoxia. However, severe forms of the disease can cause pyrexia, weakness, respiratory rates and, sometimes, abortion [2]. The remarkable detection of *T. orientalis* (49%) in this study could be explained by the abundance and wide distribution of the competent vectors of this group in the island [8]. These results agree with the previous detection of the *T. orientalis* group in several tick species (including *Haemaphysalis punctata*, *Rhipicephalus annulatus* and *Dermacentor marginatus*), aborted samples and asymptomatic and symptomatic domestic ruminants in Sardinia, suggesting that these pathogens circulate in mammalian hosts in the study area [8,9]. Therefore, cattle owners and veterinarians in Sardinia should include screening for the *T. orientalis* group in the presence of the above symptoms of unknown origin.

*T. equi*, the causative agent of equine piroplasmosis, was the only piroplasm species detected in horses from this region. Ticks belonging to *Hyalomma*, *Rhipicephalus* and *Dermacentor* genera are well-recognized vectors of *T. equi* [13]. The replication of merozoites into erytrocites, followed by the rupture of the host cells, causes hemolytic anemia, hemoglobinuria and icterus. Since these tick species are abundant in Sardinia, we suppose that their prevalence on the island should be taken as an indication of exposure to *T. equi*. Because these protozoa are widespread in high numbers of horses across various areas of Sardinia (as reported here) and were previously reported in the findings of Zobba et al. [14], further investigations are needed to confirm their distribution in the study area. 

The presence of *B. bigemina* has also been identified in three cattle from one farm located in northern Sardinia. *B. bigemina*, the agent of cattle babesiosis, is regarded as one of the most important infectious causes of severe economic losses to the cattle industry worldwide. The economic losses due to cattle babesiosis are related not only to mortality, reduced productivity and cost control measures, but also to the international cattle trade [15,16]. *R. annulatus,* one of the main vectors and reservoirs of *B. bigemina* [11], is widely distributed in areas in the south of Sardinia, and *B. bigemina* was identified in the tick species collected from these cattle [8]. Since the infectious agent mirrors the geographic distribution of its competent vector, *R. annulatus*, more investigations are necessary to further understand the epidemiology of *B. bigemina* in Sardinia.

In this study, 14% of the samples were positive for *T. annulate,* whose occurrence has never been reported in mammals from Sardinia. *T. annulata*, the aethiologic agent of tropical theileriosis in cattle [17], is considered one of the most common tick-borne pathogens affecting domestic ruminants in several countries of Asia, North Africa, the Middle East and Europe, including Italy [18,19]. The *Hyalomma* species are known as vectors of *T. annulate,* as well as ticks of the genera *Haemaphysalis,* which were recently identified as the vector of *T. annulata* in Sicily [20]. Since *Haemaphysalis* ticks are widespread in the study region, more studies are needed to determine the prevalence of these disease agents in Sardinia.

## 5. Conclusions

In this study, we expand the knowledge on piroplasms in Sardinian mammals and add the *T. annulata* species to the repertoire of piroplasms already detected in Sardinia, Italy.

Further epidemiological studies are needed to monitor the prevalence of *Theileria* and *Babesia* infections in vertebrate hosts, including those without noticeable symptoms, and evaluate the regional differences in the frequency of piroplasmid infections, also including those tick species that act as vectors. 

## Figures and Tables

**Table 1:** Number of samples tested positive for *Babesia* and *Theileria* spp. and details of sequences obtained from horses and cattle in this study.

Animal Hosts	No. of Examined Animals	No. of Infected Animals (%)	No. of Good Quality Sequences	Pathogens Sequenced (Identificative Number of Collection Site)	1st Hit GenBank Accession Number (ID%)
Cattle	72	49 (68%)	24/49	*T. orientalis/sergenti/buffeli* (13, 27, 31, 36, 44, 45)	MN611176
			7/49	*T. annulata* (17, 22, 37)	MT341858
			3/49	*B. bigemina* (7)	MH050356
Horse	80	60 (75%)	36/60	*T. equi* (2, 3, 5, 10, 16, 19, 23, 25, 28, 33, 34, 35, 36, 39, 43, 47)	MT463613

## Data Availability

Not applicable.
